# Impedance-based analysis of Natural Killer cell stimulation

**DOI:** 10.1038/s41598-018-23368-5

**Published:** 2018-03-21

**Authors:** Frank Fasbender, Carsten Watzl

**Affiliations:** Leibniz Research Centre for Working Environment and Human Factors, IfADo, TU-Dortmund, D-44139 Dortmund, Germany

## Abstract

The use of impedance-based label free cell analysis is increasingly popular and has many different applications. Here, we report that a real-time cell analyzer (RTCA) can be used to study the stimulation of Natural Killer (NK) cells. Engagement of NK cells via plate-bound antibodies directed against different activating surface receptors could be measured in real time using the label-free detection of impedance. The change in impedance was dependent on early signal transduction events in the NK cells as it was blocked by inhibitors of Src-family kinases and by inhibiting actin polymerization. While CD16 was the only receptor that could induce a strong change in impedance in primary NK cells, several activating receptors induced changes in impedance in expanded NK cells. Using PBMCs we could detect T cell receptor-mediated T cell activation and CD16-mediated NK cell activation in the same sample. Performing a dose-response analysis for the Src-family kinases inhibitor PP1 we show that T cells are more sensitive to inhibition compared to NK cells. Our data demonstrate that the RTCA can be used to detect physiological activation events in NK cells in a label-free and real-time fashion.

## Introduction

Natural killer (NK) cells are an essential part of the innate immune system. They belong to a group of cytotoxic innate lymphoid cells and are important for early and effective immune responses against cancer and virus-infected cells^1–3^. In addition, they are regulators of adaptive immune responses and also play a role in tissue homeostasis^4–6^. The activity of NK cells is regulated *via* signals from activating and inhibitory surface receptors. Self-MHC class I recognizing inhibitory receptors are important for the education of NK cells and ensure their self-tolerance. NK cell effector functions such as cellular cytotoxicity and the production of cytokines are stimulated via the engagement of different activating receptors^7^. In contrast to T- and B-lymphocytes, whose activity is critically dependent on a single antigen-specific receptor, NK cells can be activated via a variety of different germ-line encoded surface receptors. NK cell activating receptors can be grouped according to their intracellular signaling motifs. NKp30, NKp44, NKp46, and CD16 signal via an Immunoreceptor Tyrosine-based Activation Motif (ITAM); 2B4, NTB-A, and CRACC via an Immunoreceptor Tyrosine-based Switch Motif (ITSM); NKG2D and DNAM-1 signal via an Immunoreceptor Tyrosine Tail (ITT)–like motif, and NKp65 and NKp80 contain a hem-ITAM in their cytoplasmic tail^3,8^. All these activating receptors recognize different host or pathogen-derived ligands and upon ligand interaction can stimulate NK cell effector functions^3^.

To fully activate resting human NK cells, at least two distinct activating receptors have to be engaged^9^. Therefore, the term ‘co-activating receptors’ is used to describe the different activating NK cell receptors^10^. The Fc-receptor CD16 is an exception, as engagement of CD16 alone can stimulate resting human NK cells. The activity of NK cells can be enhanced by cytokines such as IL-2, IL-12, IL-15, IL-18, and IL-21^11^. Such pre-activated NK cells show stronger cytolytic activity and an enhanced ability to produce cytokines upon activation and are being utilized in immunotherapeutic approaches against cancer^12,13^. Interestingly, cytokine pre-activated NK cells are less dependent on co-activation as the engagement of individual receptors alone can stimulate effector functions by these cells^14^.

The triggering of NK cell cytotoxicity involves a number of highly regulated processes^15^. One of the first steps after the engagement of activating receptors involves the phosphorylation of Tyrosine residues in the cytoplasmic signaling domain of the receptor by Src-family kinases. This initiates a signaling network resulting in actin reorganization and inside-out signaling to enhance the binding affinity of integrins such as LFA-1^16^, which is necessary for strong adhesion to target cells and the formation of an immunological synapse^17^. Lytic granules are then recruited to this contact site and exocytosed in a regulated and directed fashion^15^, resulting in the death of the locally attached target cell. Finally, the contact is severed^18^, enabling the NK cell to kill additional targets in what is known as serial killing^19^.

Antigen receptors in T- and B-lymphocytes rely on ITAM-based signaling. While several NK cell receptors such as NKp30 or CD16 also use ITAM-based signaling adapters, there are still some differences. We could recently show that in contrast to T cells, ITAM-based receptors in NK cells rely less on the activity of Src-family kinases to initiate their signaling networks^20^. This is due to the fact that NK cells not only express the kinase ZAP-70, which is essential for T cell receptor signaling, but also the related kinase SYK, which is important for the initiation of B cell receptor signals.

The multitude of NK cell receptors, which rely on different intracellular signaling pathways, represents a challenge for the investigation of NK cell reactivity. Various assays exist to measure NK cell effector functions such as degranulation or the production of cytokines^21–23^. Impedance based-assays such as the xCELLigence system have the advantages of providing label-free, real-time measurements of cellular functions. This method has been applied to successfully measure proliferation, migration, cytotoxicity and receptor-mediated signaling^24–26^. It records changes in cell morphology, adherence and cell numbers as changes in impedance over time using specialized E-plates with gold-electrodes at the bottom of the well^27,28^. This impedance value is expressed as a dimensionless cell index (CI). A label-free and real-time measurement has many advantages over conventional endpoint assays. For example, the killing of adherent target cells by cytotoxic immune cells in suspension can be observed over time using this technology^24,29,30^. Additionally, the xCELLigence system has been described as a quick way to study receptor activation based on phenotype/morphological changes. Although typically, cells that grow in suspension culture do not influence the CI signal, previous studies successfully used this system to study T cell activation^31^ as well as mast cell activation^25,32^ via the engagement of surface receptors. Here we use an impedance-based assay system to measure NK cell responses upon the engagement of various surface receptors.

## Results

### Real-time analysis of NK cell activation events via CD16

**Figure 1:**
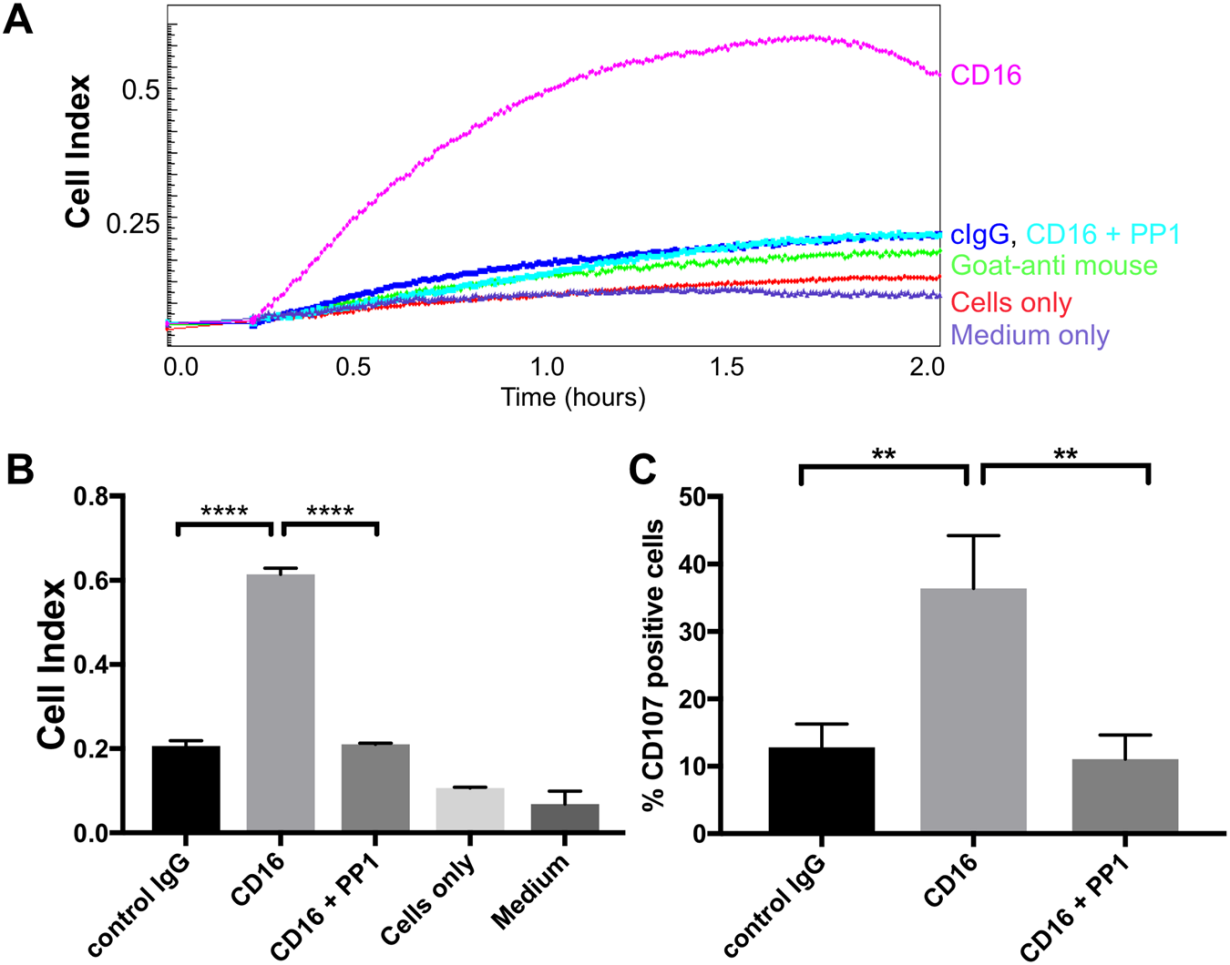
Cell Index reflects NK cell activation. (**A**) Cultured human NK cells were pre-incubated for 30 min with PP1 or DMSO as carrier control and seeded in technical triplicates onto non-coated E-plates (cells only) or on E-plates pre-coated with goat-anti-mouse antibodies in combination with control antibodies (cIgG) or antibodies directed against CD16. Cell index was recorded for 2 hours every 1 min. (**B**) Peak Cell Index values for the indicated conditions. (**C**) NK cells from (**A**) were removed from the E-plate after 2 hours and analyzed by flow cytometry for CD107a positive cells. Error bars depict technical triplicates from the same experiment and donor. The shown experiment is representative of three biological replicates.

First we wanted to test if the impedance-based xCELLigence system can detect changes in NK cells caused by the engagement of activating receptors. We decided to use plate-bound antibodies to specifically trigger defined surface receptors (see Methods and Fig. S1). Culturing NK cells on non-coated E-plates or on E-plates, which were coated with goat-anti-mouse antibodies, did not result in any significant changes of CI values compared to empty wells with medium alone (Fig. 1A). This confirms the finding that cells, which grow in suspension, do not influence the CI signal. Immobilizing an anti-CD16 antibody on goat-anti-mouse coated E-plates resulted in a rapid increase of the CI value of the cultured NK cells, reaching the maximum CI within 2 hours. The CI value increased about 3–4 fold at peak compared to E-Plates pre-coated with an isotype-control antibody (Fig. 1B). To exclude that this increase in CI is simply a result of immobilizing NK cells to the E-plates via the anti-CD16 antibody, we used the Src-family kinase inhibitor PP1. Src-family kinases are important for the phosphorylation of the ITAMs in the CD16-associated signaling chains and PP1 can therefore block CD16-mediated NK cell activation directly at the receptor level. However, PP1 does not interfere with the engagement of CD16 by the immobilized antibody. Pre-treatment of NK cells with PP1 reduced the CI in anti-CD16 coated wells to background levels (Fig. 1A,B), confirming that the change in CI is caused by specific NK cell activation events. To more directly correlate this with NK cell effector functions we removed the cells from the wells after the 2 hours incubation period and analyzed them by FACS for the degranulation marker CD107a (Fig. 1C). Cells from wells pre-coated with anti-CD16 antibody showed significantly increased degranulation compared to wells coated with isotype control antibody. PP1 effectively blocked CD16-induced degranulation, paralleling the results of the xCELLigence system. This demonstrates that we can use an impedance-based system to measure changes in NK cells induced by CD16-signaling.

**Figure 2:**
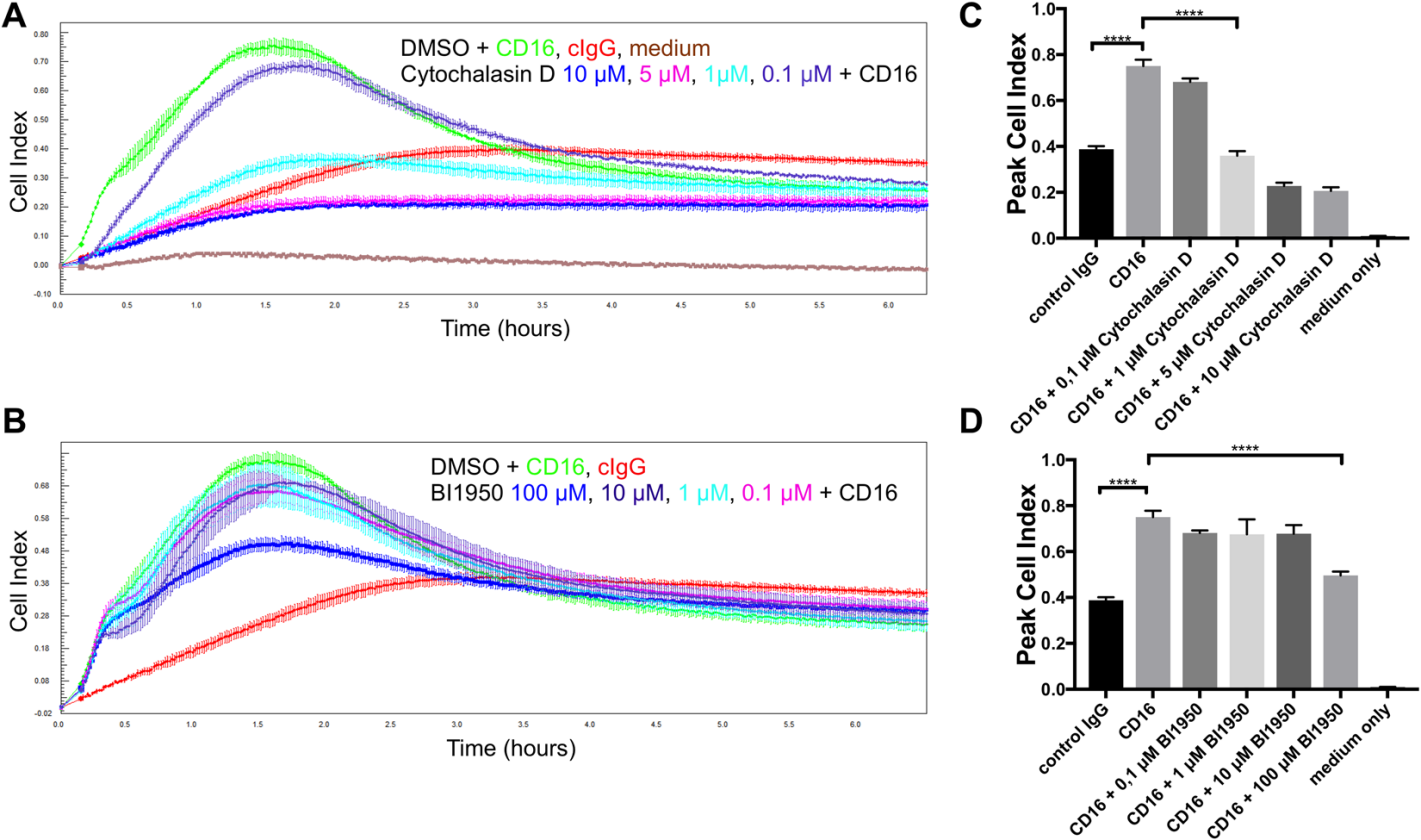
CI changes are dependent on actin polymerization but independent of LFA-1. Cultured human NK cells were pre-incubated for 30 min with (**A**,**C**) Cytochalasin D, (**B**,**D**) BI-1950 or DMSO as carrier control and seeded in technical triplicates onto E-plates pre-coated with goat-anti-mouse antibodies in combination with control antibodies (cIgG) or antibodies directed against CD16. Cell index was recorded for 6 hours every 1 min. Error bars depict technical triplicates and the shown experiment is representative of three biological replicates.

Changes in CI are typically a reflection of changes in cellular adhesion and spreading onto the E-plates. These processes are dependent on the reorganization of the actin cytoskeleton. To test if this is also true for the CD16-induced changes in CI, we treated NK cells with Cytochalasin D, an inhibitor of actin polymerization (Fig. 2A). Cytochalasin D dose-dependently inhibited the increase in CI induced by CD16 stimulation (Fig. 2C). This suggests that actin polymerization is important to induce the changes in CI, likely by inducing NK cell adhesion and spreading. In line with this we observed that PP1 could inhibit actin polymerization in CD16-stimulated NK cells (Fig. S2). The integrin LFA-1 is important for the adhesion of NK cells to target cells and the activity of integrins can be stimulated via the engagement of activating NK cell receptors^16^. We therefore tested the effect of BI-1950, potent inhibitor of the LFA-1/ICAM-1 interaction^33^, in our system (Fig. 2B). Interestingly, BI-1950 had no effect on the change in CI induced by the engagement of CD16 and only at very high concentrations we observed some inhibition. This may be explained by the fact that we do not have the LFA-1 ligand ICAM-1 in our system.

### Profiles of resting and activated NK cells

**Figure 3:**
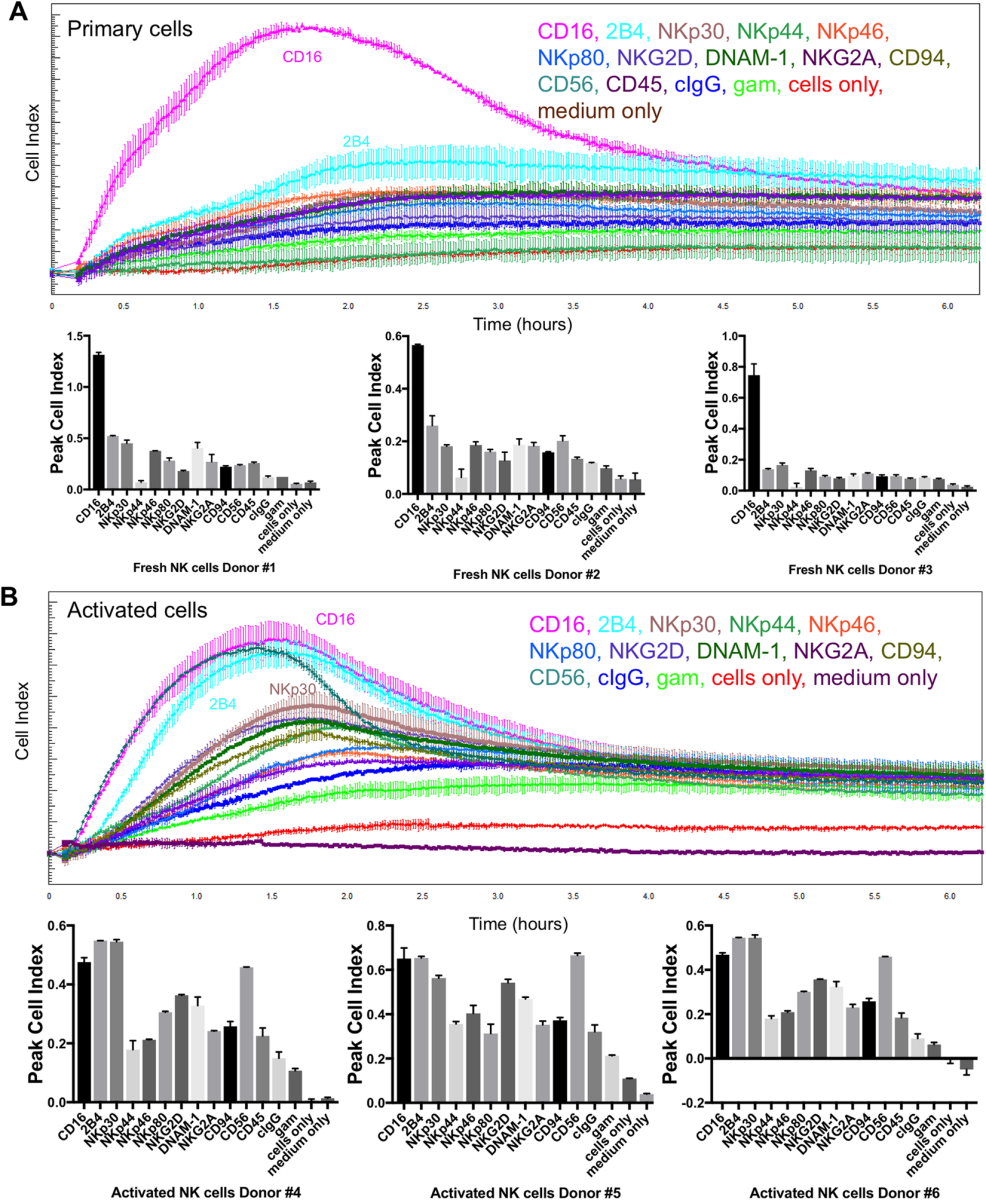
Profiles of resting and pre-activated NK cells. Equal numbers of (**A**) resting or (**B**) pre-activated NK cells from different healthy donors were seeded in duplicates onto E-Plates pre-coated with the indicated antibodies. Controls with goat anti-mouse, only cells and only medium were included. Cell index was recorded every 30 s for 6 hours. The peak cell index for each donor is shown in the bar graphs. Error bars depict technical duplicates. All biological replicates from different donors are shown and quantified.

Next we wanted to test if also the engagement of other activating NK cell receptors can be measured using the impedance-based system. For this we immobilized antibodies against CD16, NKp30, NKp44, NKp46, 2B4, NKG2D, DNAM-1, CD94, NKG2A, CD45, CD56 or an isotype control antibody on E-plates. Additional controls were wells just pre-coated with goat-anti-mouse, wells with cells only and wells with medium only. We then added either freshly isolated (resting) NK cells or NK cells that had been expanded in cultures with feeder cells and cytokines (IL-2, IL-15, IL-21) and compared the activation profiles as recorded by changes in CI over time. For resting NK cells, only CD16 was able to elicit a strong activating signal and kinetic, while all other tested antibodies did not induce a clear activating CI signal (Fig. 3A). Some receptors such as 2B4 or NKp30 showed an activating kinetic despite a low increase in CI in the resting NK cells. NKp44, which is not expressed on resting NK cells, showed a low signal comparable to the isotype control. By comparison, several activating receptors were able to induce a clear activating kinetic and increase in CI for the pre-activated NK cells (Fig. 3B), demonstrating the higher reactivity of these cells. Engagement of 2B4 and NKp30 showed an even stronger increase in CI compared to CD16 stimulation in these cells. Among the ITAM-coupled receptors, NKp30 and CD16 showed the strongest response with NKp46 and NKp44 only inducing a very small increase in CI (Fig. 3B). Triggering via NKG2D, DNAM-1, and NKp80 resulted in a clear increase in CI, albeit at a lower level compared to 2B4 or CD16. Interestingly, we observed a strong increase in CI upon engagement of CD56 in pre-activated NK cells. In contrast, engagement of CD45, which is also highly expressed by NK cells, did not result in a significant increase in CI and was comparable to the engagement of the inhibitory receptors CD94 and NKG2A. This demonstrates that the impedance-based assay system can measure NK cell changes induced by the engagement of a variety of different activating receptors.

### Co-activation and inhibition of NK cells

**Figure 4:**
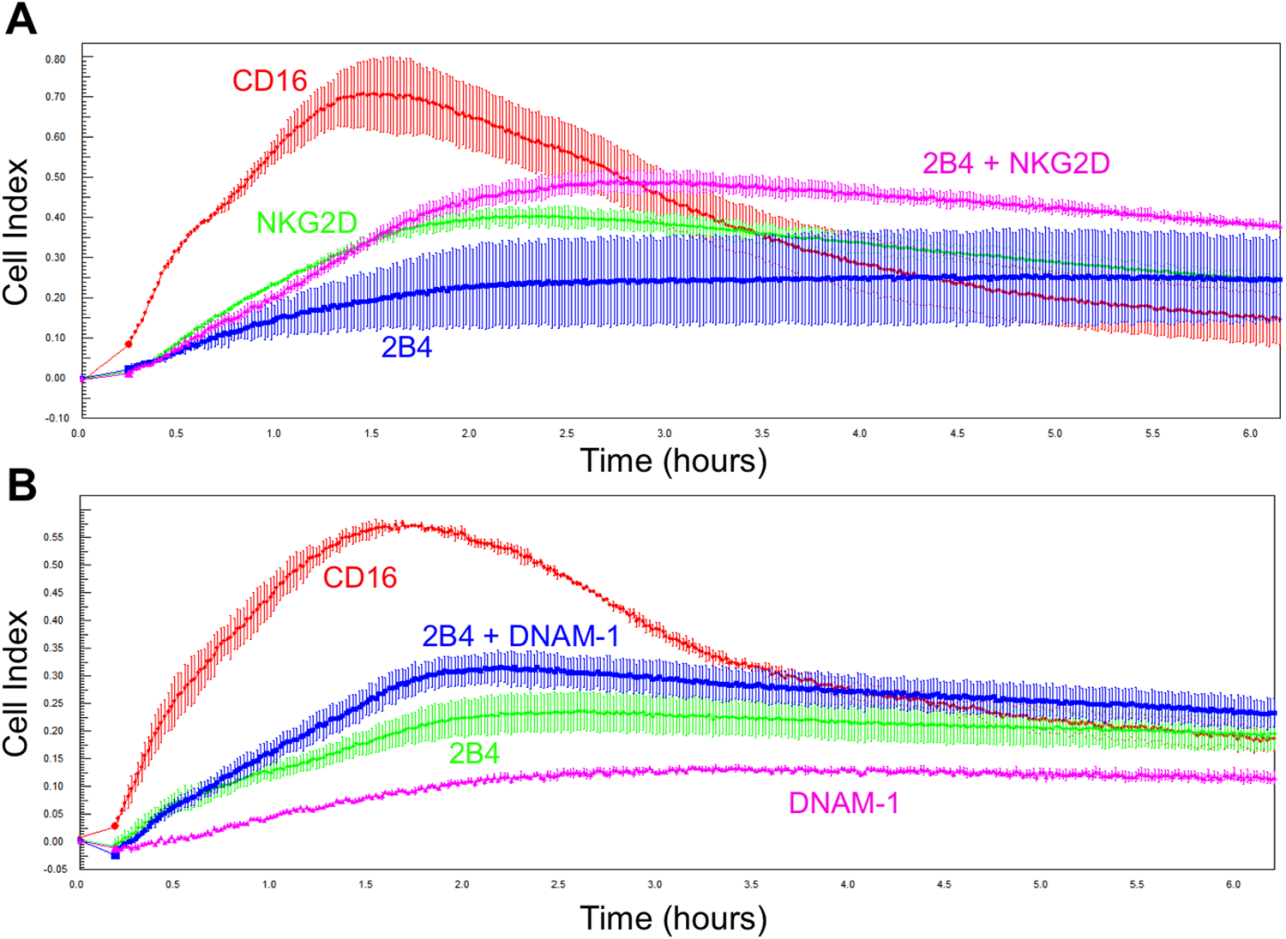
Co-activation of resting NK cells. Equal numbers of resting NK cells were seeded in technical duplicates onto E-Plates pre-coated with antibodies against the indicated receptors. Cell index was recorded every 30 s for 6 hours.

Co-activation of combinations of different receptors are necessary to fully activate resting NK cells^9^ and even pre-activated NK cells can react stronger and are more refractory to inhibitory signaling when stimulated via co-activation^14^. Therefore, we examined if co-stimulation of activating NK cell receptors further increases the CI signal. We pre-coated E-Plates with antibodies against the activating receptors 2B4 and NKG2D or DNAM-1 either alone or in combination. We found that the co-engagement of 2B4 and NKG2D or 2B4 and DNAM-1 only slightly increased the recorded CI in resting NK cells, and the response did not reach the level of CD16 stimulation (Fig. 4A,B). This suggests that the signaling events, which are enhanced by co-activation of resting NK cells, are only partially captured by measuring changes in impedance.

Inhibitory receptors can block NK cell activation by interfering with upstream signaling events of activating NK cell receptors^34^. To test if inhibitory receptors can also interfere with the increase of CI we co-engaged the inhibitory receptor NKG2A, which is expressed on a large percentage of human NK cells, together with CD16. While engagement of NKG2A by itself did not induce a significant increase in CI (Figs 3 and S3), co-engagement of NKG2A and CD16 resulted in a smaller increase of CI compared to CD16 engagement alone (Fig. S3). This demonstrates that inhibitory receptors can also interfere with CD16-mediated activation events that are detected in our system.

### T cells and NK cells show differential sensitivities to PP1

**Figure 5:**
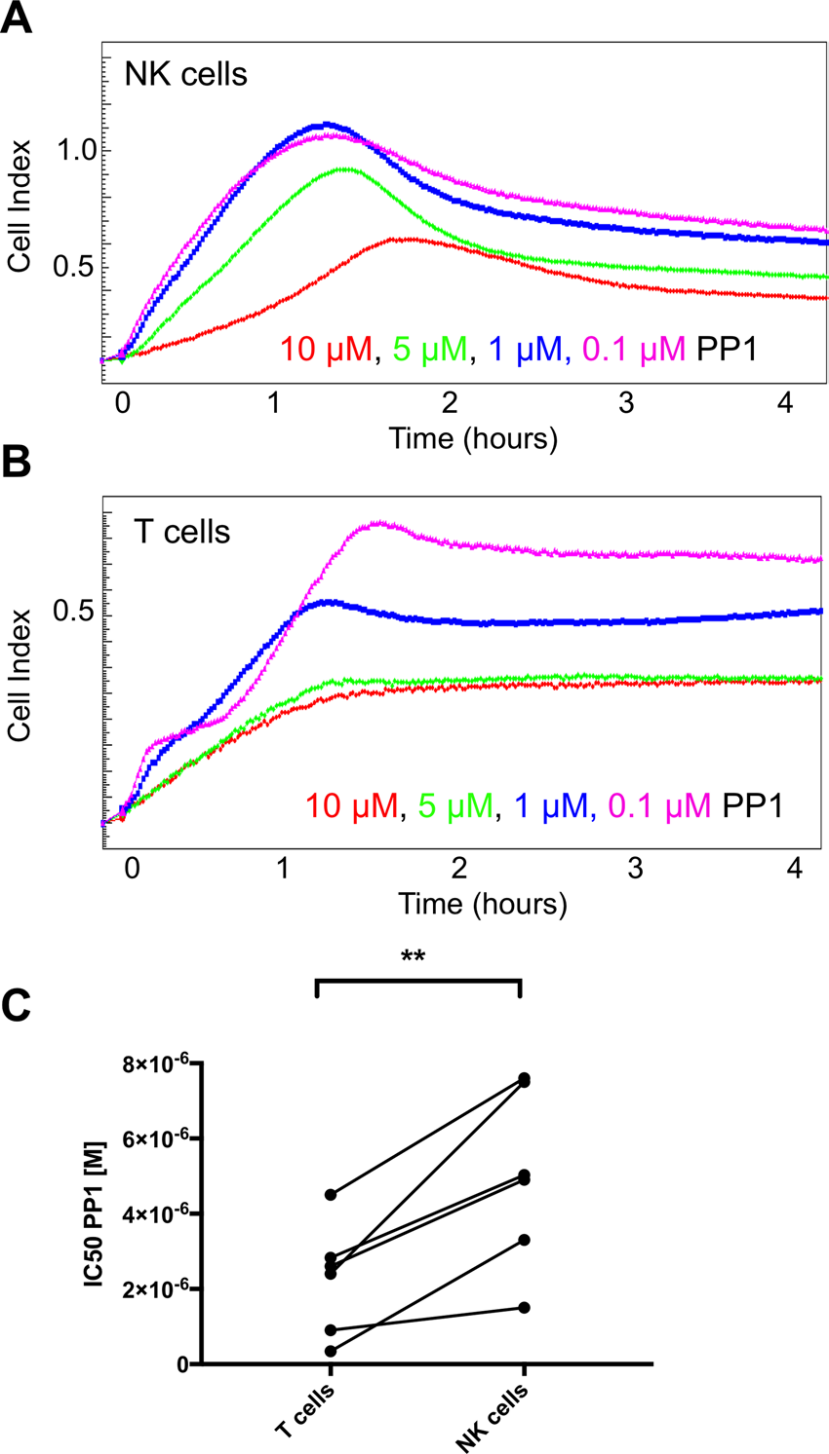
NK cells and T cells show different sensitivities for PP1. Freshly isolated PBMCs were pre-incubated with indicated concentrations of PP1 and seeded in technical duplicates on E-Plates pre-coated with antibodies against (**A**) CD16 or (**B**) CD3/CD28. CI was recorded for 6 hours every 30 s. (**C**) PP1 IC50 for T cells and NK cells was calculated using RTCA analysis software 1.0. (n = 6, p = 0.0063, Paired t-test).

As cells grown in suspension culture do not change the CI when they are not activated, we were wondering if we could determine NK or T cell activation events within a mixture of cells. Therefore, we isolated peripheral blood mononuclear cells (PBMC), which contain T cells, B cells, NK cells and monocytes. We coated E-plates with anti-CD3 and anti-CD28 antibodies to specifically stimulate T cells and we used anti-CD16 antibodies to stimulate NK cells. We had previously shown that T cell receptor-induced SLP-76 phosphorylation in T cells is more sensitive to inhibition by the Src-family kinase inhibitor PP1 compared to SLP-76 phosphorylation in NK cells upon stimulation of the ITAM-coupled receptor CD16^20^. The xCELLigence software can create dose-response curves and determine the corresponding IC50 or EC50 by fitting changes in CI over concentrations of a given compound. To compare the sensitivities of NK cells and T cells to PP1 inhibition, we stimulated PBMCs in either CD16 or CD3/CD28 pre-coated E-Plates after pre-incubation with different concentrations of PP1 or DMSO control (Fig. 5A,B). We observed a clear increase in CI when we stimulated PBMCs with CD16 or CD3/CD28, demonstrating that this system can be used to determine T and NK cell activation events even in a mixture of cells. However, we could not measure T cell or NK cell activation events in whole blood samples, as the background increase in CI was too high to detect any specific cell stimulation (Fig. S4). As expected, PP1 dose-dependently inhibited NK cell and T cell responses with a smaller and sometimes delayed response peaks (Fig. 5A,B). We calculated the IC50 at the peak of each stimulation using the xCELLigence software. We found that the IC50 of PP1 for T cells was consistently lower (1–4 μM) than for NK cells (3–7 μM) for each donor (Fig. 5C).

## Discussion

Here we describe the evaluation of a swift and label-free phenotypic assay for the assessment of NK cell activation events that is based on the xCELLigence system. Our data show that the changes measured by impedance are indeed reflective of the initiation of NK cell signaling pathways. CD16 receptor activation led to impedance changes that were significantly increased and showed a markedly different kinetic compared to isotype control treated cells. This correlated with NK cell activation as assessed by conventional methods such as degranulation. More importantly, pharmacological blockade of early signal transduction events such as Src-family kinase activity and actin reorganization suppressed the impedance response. Engagement of activating NK cell receptors leads to the reorganization of the actin cytoskeleton, resulting in NK cell polarization, and also affects the membrane organization of surface receptors^17,34–36^. These early signaling events are likely the cause for the change in impedance detected by our measurements. Indeed, an earlier study, which used an impedance-based system to measure T cell activation via the engagement of CD3 and CD28, has also described cytoskeletal rearrangement as the cause of the changes in impedance^31^. We have previously shown that the engagement of CD16, 2B4 and NKp30 resulted in a strong increase in integrin-mediated adhesion in NK cells^16^. The same receptors also gave the strongest response in the xCELLigence system. Therefore, the impedance-based system most likely detects changes in NK cell adhesion and polarization. However, as we do not have the LFA-1 ligand ICAM-1 in our system, the LFA-1 antagonist BI-1950 did not inhibit the increase in CI. Typically, engagement of activating receptors can induce NK cell adhesion and polarization within 10–30 minutes^15,16^. In our system the CI signal peaked between 1–2 h, which is significantly later. This may be explained by the fact that in our population-based measurement not all NK cells react equally fast, consistent with previous reports of great NK cell heterogeneity^37^. However, the increase in CI was also transient, suggesting that the events detected in the impedance-based system are also down-regulated after a while. This was in contrast to a more slow increase in CI under control conditions. Therefore, not only the CI peak value, but also the timing of the response is important to detect NK cell activation events.

Pre-coating with an antibody against the NK cell marker CD56 also induced a strong change in CI signal. Interestingly, this was only evident with cultured NK cells and was not observed when we used freshly isolated, resting NK cells. The kinetic of the CI increase was clearly indicative of physiological changes and not only of receptor binding. This is in line with other findings, describing physiological functions for CD56 on human NK cells. It was reported that CD56 can modulate NK cell cytotoxicity^38^, serve as a recognition receptor for pathogens^39^, and is involved in synapse formation during NK cell development^40^.

Our data show that an impedance-based system can be used to detect NK cell activation events stimulated by a wide array of activating receptors. The co-activation of resting NK cells via the engagement of 2B4 and NKG2D or 2B4 and DNAM-1 was partially recapitulated using this assay system. Co-activation has been shown to enhance Vav-1 phosphorylation via differential SLP-76 phosphorylation^41,42^. In turn, this results in enhanced actin reorganization, NK cell polarization and adhesion, which is likely detected by our measurements. Similarly, inhibitory receptors have been shown to interfere with Vav-1 phosphorylation^43^, which is likely the cause for the observed inhibitory effect of NKG2A co-engagement on CD16-induced changes in CI. This suggests that our method can detect physiological relevant changes in NK cells induced by the engagement of activating and inhibitory receptors in a time resolved fashion.

As cells grown in suspension do not influence the impedance measurement, we could detect specific NK cell and T cell activation events in a mixture of cells using PMBCs. This provides the possibility to directly compare the activity of different cells within the same sample. Additionally, the xCELLigence system is widely used for drug testing on many cell types. Here, we made use of this ability to directly compare the sensitivity of T and NK cell activation towards pharmacological inhibition of Src family kinases. The calculated IC50 of the PP1 inhibitor was consistently lower for T cells compared to NK cells, indicating that T cell receptor signaling is more sensitive to inhibition of Src family kinases than NK cell stimulation via CD16. We have previously reported a similar finding using SLP-76 phosphorylation as a readout^20^. Our confirmation of these results is another indication that the impedance-based system can detect physiological changes induced by lymphocyte activation in a quantitative manner.

Using NK cells from different healthy donors we could demonstrate slight differences in the reactivity of the NK cells towards the triggering of activating receptors such as NKp30. NKp30 can be expressed in different isoforms, which show different abilities to stimulate NK cells^44^. This could explain some of the donor-specific differences in our data. Additionally, it is established that NK cell reactivity differs between individuals and low NK cell cytotoxic activity is correlated with an increased incidence of cancer^45^. Our approach can quickly determine activation profiles of NK cells upon engagement of various receptors. It would be interesting to test if these profiles correlate with clinical parameters. While many methods already exist to assess the composition and activity of human immune cell *in vitro*^22,23,46,47^, our assay provides a fast and label-free alternative. Additionally, measuring NK cell responses by the impedance-based assay could be useful to determine defects in NK cell activity for the diagnosis of immune deficiencies.

## Methods

### NK Cell Culture

Peripheral blood mononuclear cells (PBMCs) were isolated from blood of healthy donors by Ficoll density gradient centrifugation (PAN-Biotech, Germany). All blood donors gave their informed consent and all experiments were performed in accordance with relevant guidelines and regulations. Human NK cells were purified from PBMCs using the Dynabeads Untouched Human NK Cell kit (Thermo Fisher Scientific) according to manufacturer’s instructions. For NK cell activation and expansion, purified NK cells were cultured in 96-well round-bottom plates (Nunc) with irradiated K562-mbIL15-41BBL feeder cells (kind gift from Dario Campana) in IMDM Glutamax supplemented with 10% FCS and 1% penicillin/streptomycin, IL-2 (100 U/ml, NIH Cytokine Repository) and IL-15 (5 ng/ml, PAN-Biotech). Feeder cells were added at day 0 and day 7 of the culture and medium with fresh cytokines was exchanged every 2–3 days. IL-21 (100 ng/ml, Miltenyi Biotec) was added at the first day. NK cells were used for experiments between 3–6 weeks after isolation and they were between 90 and 99% CD3^−^, CD56^+^, and NKp46^+^ as assessed by flow cytometry.

### xCELLigence

The xCELLigence system (ACEA Biosciences) is a label-free technology that quantifies cell adhesion and spreading in real time. It measures electrical impedance across a pair of gold-plated interdigitated microelectrodes on specialized 16-well E-plates. Impedance is reported as a cell index value. The CI value at each time point is calculated as CI = (Z_i_ − Z_0_)/15 Ohm, where Z_i_ is the impedance at an individual time point during the experiment and Z_0_ is the impedance at the start of the experiment. Thus CI is a self-calibrated value derived from the ratio of measured impedances. The CI is a unitless parameter.

### E-Plate-Coating

To immobilize different antibodies in a comparable way to E-plates, we decided to coat the wells first with goat-anti-mouse antibodies. Therefore, E-Plates 16 PET were pre-coated with 50 μl of 5 μg/ml goat-anti-mouse antibody for 3 hours at 37 °C and subsequently washed three times with PBS. In pilot experiments we determined that the subsequent coating with an antibody against NK cell surface receptors gave the strongest signal (Fig. S1). In contrast, binding the antibody first to the NK cells or adding the antibody at the same time as the NK cells without washing was less effective (Fig. S1). Therefore, primary antibody was added to the pre-coated wells at 2 μg/ml in a volume of 50 μl and incubated at 37 °C for 1 hour. Plates were washed three times with PBS and background reading was performed in 100 μl medium.

The following antibodies were used: CD16 (3G8), 2B4 (C1.7), NKp46 (9E2), NKp80 (5D12), CD28 (28.2), DNAM-1 (TX25), CD56 (NCAM) (all from Biolegend), CD3 (OKT3, eBioscience), NKG2D (R&D), NKp30 (p30-15, produced in our laboratory), NKp44 (p44-2, produced in our laboratory), Mouse IgG1 (MOPC-21, Sigma), CD45 (BD Transduction laboratories), NKG2A (Z199, Beckman Coulter), goat anti-mouse (dianova), and CD94 (HP-3D9, BD Bioscience).

### Inhibitors

For experiments with inhibitors, NK cells were pre-incubated with indicated concentrations of PP1 (Biomol), Cytochalasin D (Biomol), BI-1950 (opnme program of Boeringer Ingelheim) or DMSO solvent control for 20′ at 37 °C. Cells were then added without washing to the E-Plate.

### FACS

To assess NK cell degranulation, NK cells were stimulated as described before in the presence of CD107a PE-Cy5 antibody or an isotype control. After 2 hours, NK cells were removed by pipetting, washed twice and analyzed on a BD LSRFortessa. Data were analyzed by FlowJo.

### Microscopy

Cultured NK cells were seeded onto µ-Slide 8 Well (ibidi) that were pre-coated with PLL, goat-anti-mouse and 3G8 or an isotype control (MOPC-21). After 90 min, cells were fixed, permeabelized and stained with DAPI and phalloidin-AF488. Cells were visualized using the EVOS FL Cell Imaging System (Thermo Fisher Scientific).

### Statistical analysis

Dose-response curves were analyzed with RTCA analysis software 1.0 (ACEA Bioscience). The resulting EC50 values and all other data was analyzed with Prism 7 (GraphPad). Effects were tested using one-way ANOVA and a paired t-test for Fig. 5.

### Data accessibility

All data generated or analyzed during this study are included in this published article (and its Supplementary Information files).

## Electronic supplementary material

Supplementary Figures
