# Supplementary material for: Impedance-based analysis of Natural Killer cell stimulation
Source: Sci Rep. 2018 Mar 21;8:4938. doi: 10.1038/s41598-018-23368-5 (PMC5862859; doi:10.1038/s41598-018-23368-5)
Supplement: Supplementary file 1 — Supplementary Figures [file 41598_2018_23368_MOESM1_ESM.pdf]

Supplementary Information

Impedance-based analysis of Natural Killer cell stimulation

Frank Fasbender and Carsten Watzl*

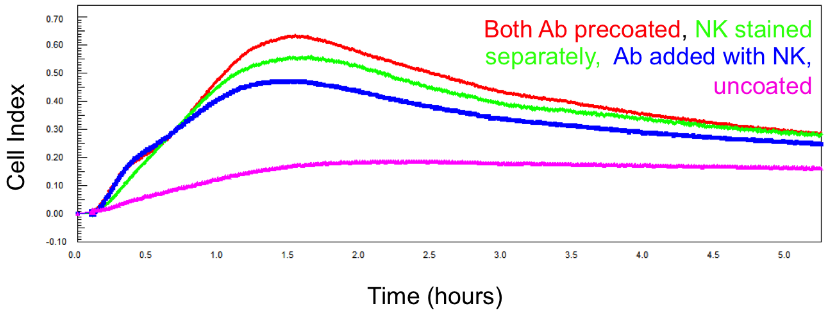

Supplementary Figure 1: Comparison of coating protocols and their effect on cell index. E-Plates were pre-coated with goat-anti mouse or left completely uncoated as control. Some wells were additionally coated with specific primary antibody (Both Ab precoated). For other wells, the NK cells were prestained with specific primary antibody (NK stained separately) or added together with the specific primary antibody (Ab added with NK). Every condition was measured in technical duplicates while the CI was recorded for 5 hours every minute.

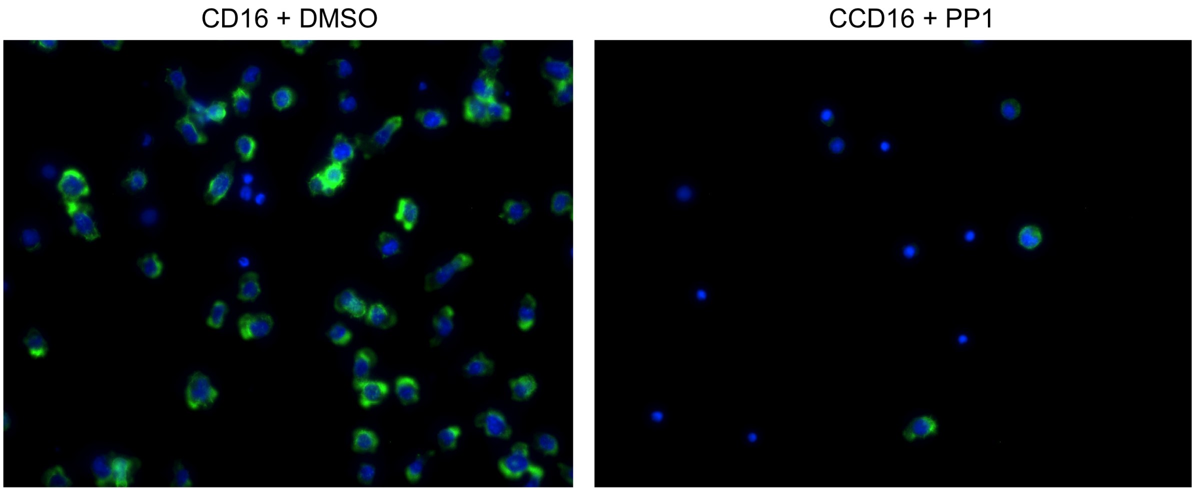

Supplementary Figure 2: Actin polymerization can be inhibited by PP1. Micro-Slides were pre-coated with goat-anti-mouse and antibodies against CD16. NK cells were pre-treated with PP1 or DMSO for 20 min. NK cells were added to the slides, incubated for 90 min, fixed and stained with DAPI (blue) and phalloidin (green).

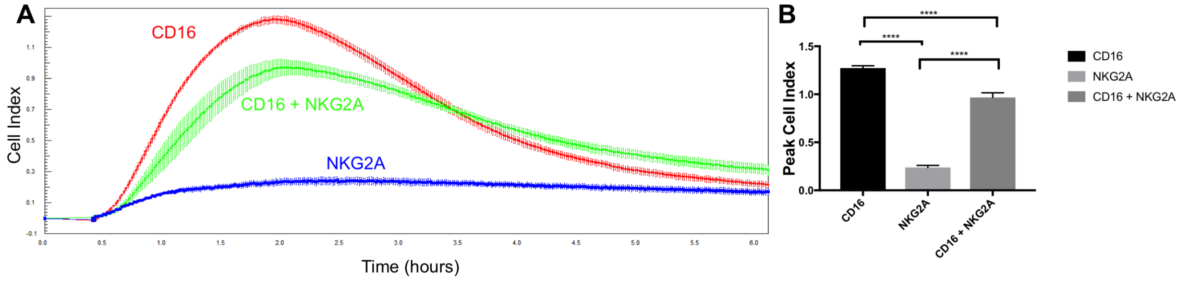

Supplementary Figure 3: CD16 activation can be inhibited by NKG2A. Freshly isolated NK cells were seeded in triplicates on E-Plates pre-coated with goat-anti-mouse and the indicated antibodies. Cell Index was recorded for 6 hours every 1 min.

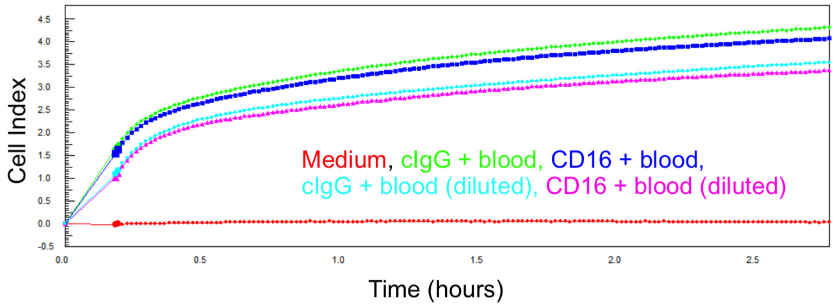

Supplementary Figure 4: Whole blood analysis. E-Plates were pre-coated with goat-anti mouse and antibodies against CD16 (3G8) or an isotype control (cIgG, MOPC-21). Heparinized whole blood was added directly or as a 1:2 dilution in PBS to the wells. Cell Index was recorded for 3 hours every 1 min.
